# 6-Isopropyl-5-meth­oxy-3-phenyl-3*H*-1,2,3-triazolo[4,5-*d*]pyrimidin-7(6*H*)-one

**DOI:** 10.1107/S1600536810041978

**Published:** 2010-10-30

**Authors:** Xiao-Hua Zeng, Hong-Mei Wang, Shou-Heng Deng, Li-Li Chen

**Affiliations:** a Institute of Medicinal Chemistry, Hubei Medical University, Shiyan 442000, People’s Republic of China; b Center of Oncology, People’s Hospital affiliated with Hubei Medical University, Shi Yan 442000, People’s Republic of China

## Abstract

In the title compound, C_14_H_15_N_5_O_2_, the whole mol­ecule apart from the terminal C atoms of the isopropyl group is located on a crystallographic mirror plane. An intra­molecular C—H⋯N hydrogen-bonding inter­action may stabilize the mol­ecular conformation. The crystal packing features weak slipped π–π inter­actions between the pyrimidine and the phenyl rings of symmetry-related mol­ecules [centroid–centroid distance = 3.746 (1)Å, slippage of 1.574 Å].

## Related literature

Fpr the biological activity of 8-aza­guanine derivatives, see: Roblin *et al.* (1945 ▶); Ding *et al.* (2004 ▶); Mitchell *et al.* (1950 ▶); Levine *et al.* (1963 ▶); Montgomery *et al.* (1962 ▶); Yamamoto *et al.* (1967 ▶); Bariana (1971 ▶); Holland *et al.* (1975 ▶). For related structures, see: Chen & Shi (2006 ▶); Ferguson *et al.* (1998 ▶); Li *et al.* (2004 ▶); Maldonado *et al.* (2006 ▶); Wang *et al.* (2006 ▶); Xiao & Shi (2007 ▶); Zeng *et al.* (2006 ▶, 2009 ▶); Zhao, Hu *et al.* (2005 ▶); Zhao, Wang & Ding (2005 ▶); Zhao, Xie *et al.* (2005 ▶).
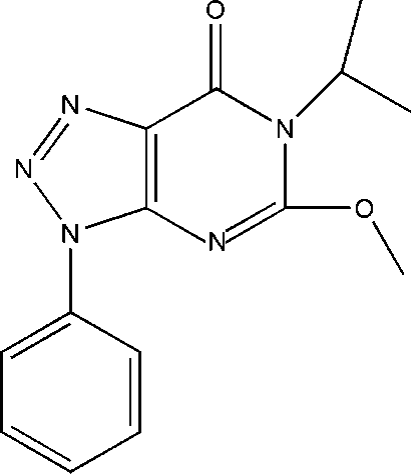


## Experimental

### 

#### Crystal data

C_14_H_15_N_5_O_2_
*M* *_r_* = 285.31
Orthorhombic, 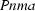

*a* = 14.921 (2) Å
*b* = 6.7989 (11) Å
*c* = 13.839 (2) Å
*V* = 1404.0 (4) Å^3^
*Z* = 4
Mo *K*α radiation
μ = 0.10 mm^−1^
*T* = 298 K
0.16 × 0.12 × 0.10 mm

#### Data collection

Bruker SMART CCD area-detector diffractometer
Absorption correction: multi-scan (*SADABS*; Sheldrick, 2008 ▶) *T*_min_ = 0.985, *T*_max_ = 0.991
7422 measured reflections
1418 independent reflections
1045 reflections with *I* > 2σ(*I*)
*R*_int_ = 0.039

#### Refinement

*R*[*F* ^2^ > 2σ(*F* ^2^)] = 0.065
*wR*(*F* ^2^) = 0.185
*S* = 1.07
1418 reflections
125 parameters
H-atom parameters constrained
Δρ_max_ = 0.23 e Å^−3^
Δρ_min_ = −0.29 e Å^−3^

### 

Data collection: *SMART* (Bruker, 2001 ▶); cell refinement: *SAINT* (Bruker, 2001 ▶); data reduction: *SAINT*; program(s) used to solve structure: *SHELXS97* (Sheldrick, 2008 ▶); program(s) used to refine structure: *SHELXL97* (Sheldrick, 2008 ▶); molecular graphics: *ORTEPIII* (Burnett & Johnson, 1996 ▶), *ORTEP-3 for Windows* (Farrugia, 1999 ▶) and *PLATON* (Spek, 2009 ▶); software used to prepare material for publication: *SHELXTL* (Sheldrick, 2008 ▶).

## Supplementary Material

Crystal structure: contains datablocks global, I. DOI: 10.1107/S1600536810041978/dn2610sup1.cif

Structure factors: contains datablocks I. DOI: 10.1107/S1600536810041978/dn2610Isup2.hkl

Additional supplementary materials:  crystallographic information; 3D view; checkCIF report

## Figures and Tables

**Table 1:** Hydrogen-bond geometry (Å, °)

*D*—H⋯*A*	*D*—H	H⋯*A*	*D*⋯*A*	*D*—H⋯*A*
C2—H2⋯N4	0.93	2.37	3.021 (4)	127

**Fig. 1.**
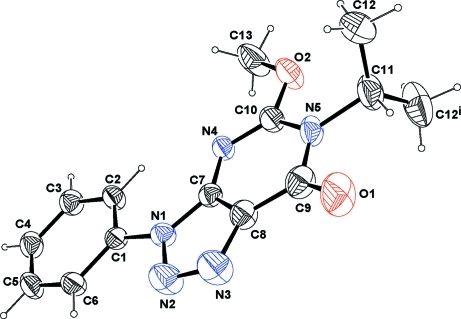
View of the molecule of showing the atom-labeling scheme. Displacement ellipsoids are drawn at the 30% probability level. H-atoms are reprsented as small spheres of arbitrary radii. [Symmetry code: (i) -x-1/2, y+1/2, z-1/2].

**Fig. 2.**
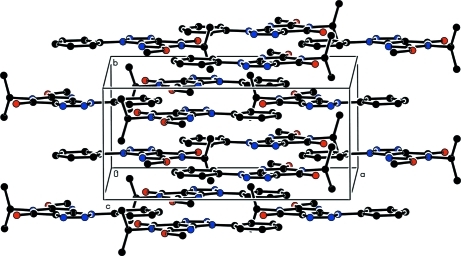
Packing view showing the stacking of the molecules along the b axis. H atoms have been omitted for clarity.

## supplementary crystallographic information

### Comment

The derivatives of heterocycles containing 8-azaguanine system, which are well known bioisosteres of guanine, are of great importance because of their remarkable biological properties, such as antimicrobial or antifungal activities (Roblin *et al.*, 1945; Ding *et al.*, 2004), encephaloma cell inhibitor (Mitchell *et al.*, 1950; Levine *et al.*, 1963), antileukemie (Montgomery *et al.*, 1962), hypersusceptibility inhibitor and acesodyne activities (Yamamoto *et al.*, 1967; Bariana, 1971; Holland *et al.*, 1975).

In recent years, Zhao's group succeeded in synthesizing the derivatives of 8-azaguanine *via* aza-Wittig reaction of beta-ethoxycarbonyl iminophosphorane with aromatic isocyanates (Zhao, Xie *et al.*, 2005). As a continuation of the quest for new biologically active derivatives of 8-azaguanine, the title compound, (I), was obtained from beta-ethoxycarbonyl iminophosphorane with aliphatic isocyanate, and structurally characterized.

In the title compound, C_14_H_15_N_5_O_2_, the whole molecule but the terminal C atoms of the isopropyl group is located in a mirror plane and is then perfectly planar (Fig. 1). The bond lengths and angles in the triazolopyrimidinone moiety are in good agreement with those observed for closely related structures (Zhao, Hu *et al.*, 2005; Zhao, Wang & Ding, 2005). the triazolopyrimidine ring system is perfectly coplanar (Chen & Shi, 2006; Ferguson *et al.*, 1998; Li *et al.*, 2004; Maldonado *et al.*, 2006; Wang *et al.*, 2006; Xiao & Shi, 2007; Zeng *et al.*, 2009).

The molecules are packed along the b axis with weak slippest π–π interaction between the pyrimidin and the phenyl rings of symmetry related molecules (Centroid to centroid distance= 3.746 (1)Å, interplanar distance= 3.399 Å with a slippage of 1.574 Å).

### Experimental

To the solution of carbodiimide prepared according to Zeng *et al.* (2006) in a mixed solvent (CH_2_Cl_2_/MeOH,1:4 *v*/*v*, 15 ml) was added a fresh prepared solution of Na/MeOH (0.1 g/2 ml). After stirring  the reaction mixture for 6 h, the solvent was removed under reduced pressure and the residue was recrystallized from EtOH to give the title compound (I) in 89% yield (m.p. 471 K). Elemental analysis: calculated for C_14_H_15_N_5_O_2_: C, 58.94; H, 5.30; N, 24.55%. Found: C, 57.62; H, 5.72; N, 24.01%. Crystals suitable for X-ray diffraction study were obtained by recrystallization from hexane and dichloromethane (1:3 *v*/*v*) at room temperature.

### Refinement

All H atoms attached to C atoms and N atom were fixed geometrically and treated as riding with C—H = 0.98 Å (methine), 0.96 Å (methyl) or 0.93 Å (aromatic) with U_iso_(H) = 1.2U_eq_(C) or U_iso_(H) = 1.5U_eq_(C_methyl_).

### Crystal data

**Table:**

C_14_H_15_N_5_O_2_	*F*(000) = 600
*M**_r_* = 285.31	*D*_x_ = 1.350 Mg m^−^^3^
Orthorhombic, *P**n**m**a*	Mo *K*α radiation, λ = 0.71073 Å
Hall symbol: -P 2ac 2n	Cell parameters from 982 reflections
*a* = 14.921 (2) Å	θ = 2.9–20.5°
*b* = 6.7989 (11) Å	µ = 0.10 mm^−^^1^
*c* = 13.839 (2) Å	*T* = 298 K
*V* = 1404.0 (4) Å^3^	Block, colourless
*Z* = 4	0.16 × 0.12 × 0.10 mm

### Data collection

**Table:**

Bruker SMART CCD area-detector diffractometer	1418 independent reflections
Radiation source: fine-focus sealed tube	1045 reflections with *I* > 2σ(*I*)
graphite	*R*_int_ = 0.039
φ and ω scans	θ_max_ = 25.5°, θ_min_ = 2.0°
Absorption correction: multi-scan (*SADABS*; Sheldrick, 2008)	*h* = −17→18
*T*_min_ = 0.985, *T*_max_ = 0.991	*k* = −8→8
7422 measured reflections	*l* = −13→16

### Refinement

**Table:**

Refinement on *F*^2^	Primary atom site location: structure-invariant direct methods
Least-squares matrix: full	Secondary atom site location: difference Fourier map
*R*[*F*^2^ > 2σ(*F*^2^)] = 0.065	Hydrogen site location: inferred from neighbouring sites
*wR*(*F*^2^) = 0.185	H-atom parameters constrained
*S* = 1.07	*w* = 1/[σ^2^(*F*_o_^2^) + (0.0883*P*)^2^ + 0.4734*P*] where *P* = (*F*_o_^2^ + 2*F*_c_^2^)/3
1418 reflections	(Δ/σ)_max_ < 0.001
125 parameters	Δρ_max_ = 0.23 e Å^−^^3^
0 restraints	Δρ_min_ = −0.29 e Å^−^^3^

### Special details

**Table:**

Geometry. All esds (except the esd in the dihedral angle between two l.s. planes) are estimated using the full covariance matrix. The cell esds are taken into account individually in the estimation of esds in distances, angles and torsion angles; correlations between esds in cell parameters are only used when they are defined by crystal symmetry. An approximate (isotropic) treatment of cell esds is used for estimating esds involving l.s. planes.
Refinement. Refinement of *F*^2^ against ALL reflections. The weighted *R*-factor *wR* and goodness of fit *S* are based on *F*^2^, conventional *R*-factors *R* are based on *F*, with *F* set to zero for negative *F*^2^. The threshold expression of *F*^2^ > σ(*F*^2^) is used only for calculating *R*-factors(gt) *etc*. and is not relevant to the choice of reflections for refinement. *R*-factors based on *F*^2^ are statistically about twice as large as those based on *F*, and *R*- factors based on ALL data will be even larger.

### Fractional atomic coordinates and isotropic or equivalent isotropic displacement parameters (Å^2^)

**Table:**

	*x*	*y*	*z*	*U*_iso_*/*U*_eq_	Occ. (<1)
O1	0.36483 (19)	0.2500	0.3850 (3)	0.1092 (13)	
O2	0.24610 (18)	0.2500	0.6874 (2)	0.0829 (9)	
N1	0.06296 (18)	0.2500	0.41475 (18)	0.0507 (7)	
N2	0.0868 (3)	0.2500	0.3193 (2)	0.0843 (12)	
N3	0.1730 (3)	0.2500	0.3126 (2)	0.0908 (12)	
N4	0.14519 (17)	0.2500	0.5659 (2)	0.0495 (7)	
N5	0.30395 (19)	0.2500	0.5368 (3)	0.0629 (9)	
C1	−0.0298 (2)	0.2500	0.4402 (2)	0.0462 (8)	
C2	−0.0554 (2)	0.2500	0.5362 (2)	0.0546 (9)	
H2	−0.0124	0.2500	0.5848	0.065*	
C3	−0.1452 (2)	0.2500	0.5591 (3)	0.0599 (10)	
H3	−0.1626	0.2500	0.6236	0.072*	
C4	−0.2092 (3)	0.2500	0.4886 (3)	0.0630 (10)	
H4	−0.2697	0.2500	0.5049	0.076*	
C5	−0.1835 (3)	0.2500	0.3938 (3)	0.0672 (11)	
H5	−0.2270	0.2500	0.3457	0.081*	
C6	−0.0949 (3)	0.2500	0.3683 (3)	0.0580 (10)	
H6	−0.0783	0.2500	0.3035	0.070*	
C7	0.1382 (2)	0.2500	0.4688 (2)	0.0464 (8)	
C8	0.2070 (2)	0.2500	0.4039 (3)	0.0608 (10)	
C9	0.2979 (3)	0.2500	0.4346 (3)	0.0721 (11)	
C10	0.2277 (2)	0.2500	0.5942 (3)	0.0589 (9)	
C11	0.3973 (3)	0.2500	0.5789 (4)	0.0879 (14)	
H11	0.4338	0.2500	0.5200	0.105*	
C12	0.4225 (2)	0.0624 (6)	0.6212 (3)	0.1210 (15)	
H12A	0.4861	0.0599	0.6319	0.181*	
H12B	0.4063	−0.0421	0.5780	0.181*	
H12C	0.3919	0.0454	0.6816	0.181*	
C13	0.1714 (3)	0.2500	0.7534 (3)	0.1016 (17)	
H13A	0.1933	0.2500	0.8186	0.152*	
H13B	0.1356	0.1347	0.7427	0.152*	0.50
H13C	0.1356	0.3653	0.7427	0.152*	0.50

### Atomic displacement parameters (Å^2^)

**Table:**

	*U*^11^	*U*^22^	*U*^33^	*U*^12^	*U*^13^	*U*^23^
O1	0.0561 (19)	0.156 (3)	0.115 (3)	0.000	0.0314 (18)	0.000
O2	0.0545 (17)	0.125 (3)	0.0687 (18)	0.000	−0.0150 (14)	0.000
N1	0.0539 (18)	0.0584 (17)	0.0399 (14)	0.000	0.0009 (13)	0.000
N2	0.075 (2)	0.131 (3)	0.0470 (19)	0.000	0.0067 (17)	0.000
N3	0.074 (3)	0.142 (4)	0.056 (2)	0.000	0.0142 (18)	0.000
N4	0.0419 (16)	0.0507 (16)	0.0559 (18)	0.000	−0.0071 (12)	0.000
N5	0.0365 (16)	0.0593 (19)	0.093 (2)	0.000	−0.0019 (15)	0.000
C1	0.050 (2)	0.0395 (17)	0.0485 (19)	0.000	−0.0052 (15)	0.000
C2	0.043 (2)	0.066 (2)	0.055 (2)	0.000	−0.0077 (16)	0.000
C3	0.052 (2)	0.065 (2)	0.063 (2)	0.000	0.0000 (17)	0.000
C4	0.047 (2)	0.060 (2)	0.082 (3)	0.000	−0.0074 (19)	0.000
C5	0.054 (2)	0.064 (2)	0.083 (3)	0.000	−0.030 (2)	0.000
C6	0.067 (3)	0.056 (2)	0.051 (2)	0.000	−0.0148 (18)	0.000
C7	0.048 (2)	0.0419 (18)	0.0497 (19)	0.000	−0.0003 (15)	0.000
C8	0.050 (2)	0.072 (2)	0.060 (2)	0.000	0.0094 (17)	0.000
C9	0.060 (3)	0.078 (3)	0.078 (3)	0.000	0.016 (2)	0.000
C10	0.055 (2)	0.055 (2)	0.067 (2)	0.000	−0.0120 (19)	0.000
C11	0.045 (2)	0.086 (3)	0.133 (4)	0.000	−0.013 (2)	0.000
C12	0.082 (2)	0.118 (3)	0.163 (4)	0.011 (2)	−0.034 (2)	0.043 (3)
C13	0.079 (3)	0.172 (5)	0.054 (2)	0.000	−0.016 (2)	0.000

### Geometric parameters (Å, °)

**Table:**

O1—C9	1.212 (5)	C3—H3	0.9300
O2—C10	1.319 (4)	C4—C5	1.367 (6)
O2—C13	1.441 (5)	C4—H4	0.9300
N1—C7	1.350 (4)	C5—C6	1.369 (5)
N1—N2	1.368 (4)	C5—H5	0.9300
N1—C1	1.428 (4)	C6—H6	0.9300
N2—N3	1.290 (5)	C7—C8	1.364 (5)
N3—C8	1.360 (5)	C8—C9	1.421 (5)
N4—C10	1.293 (4)	C11—C12^i^	1.453 (4)
N4—C7	1.348 (4)	C11—C12	1.453 (4)
N5—C10	1.387 (5)	C11—H11	0.9800
N5—C9	1.418 (5)	C12—H12A	0.9600
N5—C11	1.510 (5)	C12—H12B	0.9600
C1—C2	1.382 (5)	C12—H12C	0.9600
C1—C6	1.391 (4)	C13—H13A	0.9600
C2—C3	1.377 (5)	C13—H13B	0.9600
C2—H2	0.9300	C13—H13C	0.9600
C3—C4	1.365 (5)		
			
C10—O2—C13	117.3 (3)	N4—C7—C8	126.8 (3)
C7—N1—N2	108.6 (3)	N1—C7—C8	105.1 (3)
C7—N1—C1	132.0 (3)	N3—C8—C7	109.4 (3)
N2—N1—C1	119.4 (3)	N3—C8—C9	129.3 (4)
N3—N2—N1	109.2 (3)	C7—C8—C9	121.4 (4)
N2—N3—C8	107.8 (3)	O1—C9—N5	120.8 (4)
C10—N4—C7	112.0 (3)	O1—C9—C8	128.1 (4)
C10—N5—C9	121.2 (3)	N5—C9—C8	111.1 (3)
C10—N5—C11	122.4 (4)	N4—C10—O2	119.6 (3)
C9—N5—C11	116.4 (3)	N4—C10—N5	127.5 (4)
C2—C1—C6	119.6 (3)	O2—C10—N5	112.9 (3)
C2—C1—N1	120.4 (3)	C12^i^—C11—C12	122.8 (5)
C6—C1—N1	120.0 (3)	C12^i^—C11—N5	113.2 (2)
C3—C2—C1	119.4 (3)	C12—C11—N5	113.2 (2)
C3—C2—H2	120.3	C12^i^—C11—H11	101.0
C1—C2—H2	120.3	C12—C11—H11	101.0
C4—C3—C2	121.1 (4)	N5—C11—H11	101.0
C4—C3—H3	119.5	C11—C12—H12A	109.5
C2—C3—H3	119.5	C11—C12—H12B	109.5
C3—C4—C5	119.3 (4)	H12A—C12—H12B	109.5
C3—C4—H4	120.3	C11—C12—H12C	109.5
C5—C4—H4	120.3	H12A—C12—H12C	109.5
C4—C5—C6	121.2 (3)	H12B—C12—H12C	109.5
C4—C5—H5	119.4	O2—C13—H13A	109.5
C6—C5—H5	119.4	O2—C13—H13B	109.5
C5—C6—C1	119.4 (3)	H13A—C13—H13B	109.5
C5—C6—H6	120.3	O2—C13—H13C	109.5
C1—C6—H6	120.3	H13A—C13—H13C	109.5
N4—C7—N1	128.1 (3)	H13B—C13—H13C	109.5

Symmetry codes: (i) *x*, −*y*+1/2, *z*.

### Hydrogen-bond geometry (Å, °)

**Table:**

*D*—H···*A*	*D*—H	H···*A*	*D*···*A*	*D*—H···*A*
C2—H2···N4	0.93	2.37	3.021 (4)	127
C6—H6···N2	0.93	2.47	2.794 (5)	100
C11—H11···O1	0.98	2.13	2.727 (7)	117
C12—H12C···O2	0.96	2.58	3.065 (5)	111
